# Is radiographic osteoarthritis associated with pain and disability of the ankle?^☆^

**DOI:** 10.1016/j.ocarto.2023.100383

**Published:** 2023-07-07

**Authors:** S.E. Kloprogge, N. Katier, A.K.E. Mailuhu, J. van Vooren, J.M. van Ochten, P.J.E. Bindels, S.M.A. Bierma-Zeinstra, M. van Middelkoop

**Affiliations:** a Department of General Practice, Erasmus MC Medical University Centre Rotterdam, Rotterdam, the Netherlands; b Department of Radiology, Albert Schweitzer Hospital, Dordrecht, the Netherlands

**Keywords:** Ankle, Osteoarthritis, Radiography, Symptoms, Chronic

## Abstract

**Objective:**

Ankle osteoarthritis (OA) is relatively understudied. It often affects younger people compared to OA in other joints. Evidence on the association between radiographic OA and ankle symptoms remains contradicting. We therefore examined the association of degree of radiographic talocrural, subtalar and talonavicular OA with severity of ankle pain, disability and predominant symptoms.

**Method:**

A cross-sectional study was conducted in a radiology department serving primary and secondary care. From the total study population (adults referred for ankle radiography), patients with chronic ankle complaints were selected (N ​= ​231). Before radiography, participants completed a questionnaire on severity of ankle pain and disability using the Ankle Osteoarthritis Scale (AOS), and on their predominant symptoms, i.e. pain, functional loss, stiffness and/or instability. To assess the associations of the Kellgren-Lawrence scores (0, 1 or ≥2) with the primary outcomes (AOS), linear regression, and with the secondary outcomes predominant symptoms, logistic regression analyses were applied.

**Results:**

Radiographic OA was not associated with AOS-pain and -disability. Radiographic talocrural OA was associated with functional loss (OR 3.26, 95% CI: 1.31; 8.11). A positive trend was seen between radiographic talonavicular OA and stiffness (OR 2.63, 95% CI: 0.97; 7.15).

**Conclusion:**

The presence of radiographic OA is not associated with severity of ankle pain and disability in patients with chronic ankle complaints referred for ankle radiography. However, radiographic talocrural OA is associated with functional loss and radiographic talonavicular OA with stiffness as predominant symptom. These findings may contribute to better recognition of ankle OA in clinical practice.

## 1 Introduction

So far, ankle osteoarthritis (OA) is relatively understudied, as research on OA generally focusses on the knee, hip and hand joints [1,2]. However, a reasonable percentage of patients with chronic ankle complaints due to trauma suffer from radiographic OA (11.7% talocrural and 4.3% talonavicular OA) [3]. Moreover, in a population referred for ankle radiography due to chronic ankle complaints, the prevalence of radiographic OA was 16% in the talocrural and 12.2% in the talonavicular joint [4]. It is important to determine the association between symptoms and radiographic OA specifically in the ankle, as ankle OA differs from OA in other joints. First of all because a relatively large percentage of ankle OA is posttraumatic and occurring in younger people [[5], [6], [7]]. Second, because radiographic ankle OA is associated with male sex, unlike OA in the knee and hand [[8], [9], [10], [11], [12]].

Contrary to the knee, only few studies examined the association between radiographic OA and ankle symptoms [[13], [14], [15]]. Within the field of knee OA, the evidence on the association between radiographic OA and symptoms has been conflicting, but stronger associations have been found in study populations with more severe knee OA [[16], [17], [18], [19], [20]]. Based on extensive research and expert opinions, different diagnostic criteria for the clinical diagnosis of knee OA (i.e. the NICE, EULAR and ACR) have been proclaimed which consist of pain, morning stiffness (<30 ​min), and functional limitation [[21], [22], [23]]. Another suggested key symptom for clinical OA is instability [24]. Remarkably, diagnostic criteria for ankle OA are still lacking [25]. Previous research has shown that radiographic ankle OA is associated with ‘presence of ankle pain, aching or stiffness’ in a community-based population [10] and with severity of pain and disability in a population with posttraumatic ankle OA [14], but not with persistent complaints after lateral ankle sprains in primary care [3].

Ankle OA related symptoms have been defined differently across studies [1,10,14,15]. For instance, studies used ‘pain in or around the foot within the last year’ [1], or ‘pain, aching or stiffness in most days of any month in the last years’ to define ankle OA related symptoms [10]. The severity of ankle OA related symptoms can be assessed using the Ankle Osteoarthritis Scale (AOS), a self-assessment instrument for pain and disability with excellent validity and reliability in isolated unilateral ankle OA [26]. In a community based study, participants with chronic ankle complaints (93% reported both pain and stiffness) had significantly lower AOS-scores and Quality of Life compared with asymptomatic participants in the control group [27], emphasizing the need to address chronic ankle complaints. Despite previous studies, the association between radiographic ankle OA and the AOS has not yet been examined in a population referred for radiography due to chronic ankle complaints. Moreover, radiographic ankle OA has not yet been examined in relation to presence of different symptoms either.

Different grading systems of radiographic ankle OA have been applied in literature. Some studies based their radiographic grading on osteophytes and joint space narrowing [1,9,10,15,28], while others also considered sclerosis, subchondral cysts [3,4,14,29], and talar tilt [14]. The joints considered varied as well; from only the tibiotalar [9,10]or talocrural joint [14] to including the subtalar joint [15] and talonavicular joint [4,29]. Signs of OA on MRI in the subtalar and talonavicular joint have been related to previous lateral ankle sprains [29]. Whether radiographic OA in these joints are associated with symptoms in patients with chronic ankle complaints remains unknown. Moreover, it is unknown whether radiographic OA features in the talocrural joint are associated with different ankle symptoms compared to the subtalar and talonavicular joint.

Therefore, our aim was to study the association between degree of radiographic talocrural, subtalar and talonavicular OA, and severity of ankle pain and disability in a population with chronic ankle complaints. We additionally explored the associations between degree of radiographic OA and predominant symptoms, i.e. pain, functional loss, stiffness and instability.

## 2 Method

### 2.1 Study design and setting

A cross-sectional study was conducted (2017–2018) in the radiology department of a secondary care hospital (Albert Schweitzer Hospital, Dordrecht, The Netherlands), serving patients referred from regional primary and secondary care. Adult patients referred for ankle radiography obtained an information letter and informed consent form for study participation. If participants gave informed consent, they completed a paper questionnaire in the waiting room before radiography. A local medical ethic science committee of the Albert Schweitzer hospital (‘Wetenschappelijk Onderzoek Advies Commissie’) approved this study (trial number 2017.11).

### 2.2 Participants

**Fig. 1:**
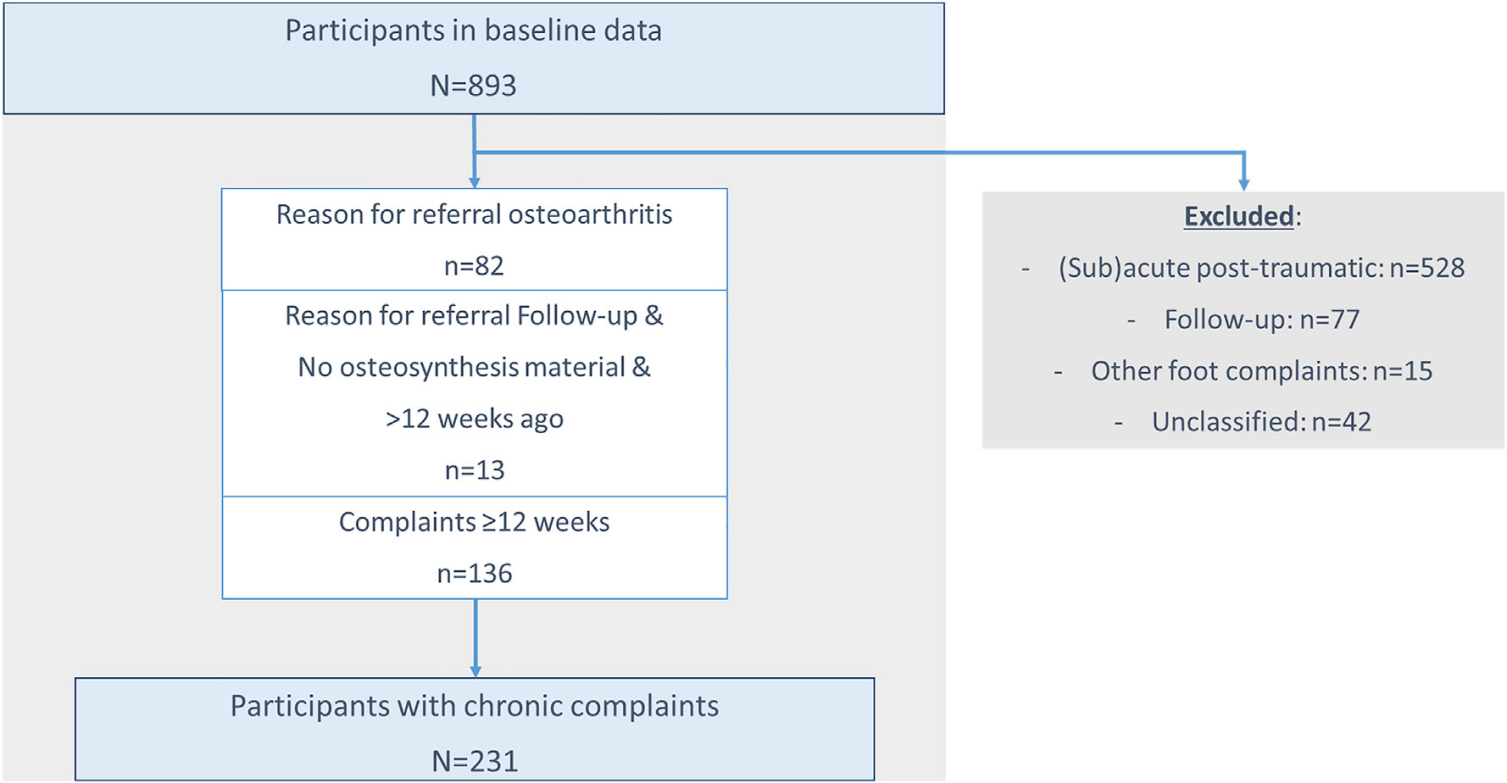
Flow chart of participants with chronic ankle complaints selected for this study.

For the current study purpose, we selected only participants with chronic ankle complaints from the total cohort study population (N ​= ​893) (Fig. 1).

To select those participants, we used the referral registry, questionnaires and information about presence of osteosynthesis material as follows: participants with OA as reason for referral, with ankle complaints for ≥12 weeks [30], without a trauma <12 weeks before inclusion, and absence of osteosynthesis material were selected for the current study. Participants with chronic ankle complaints were subsequently categorized as posttraumatic if a trauma was mentioned in the referral letter, or reported in the questionnaire.

### 2.3 Questionnaire

The paper questionnaire contained questions about demographics (i.e. age, sex, height and weight), history of ankle trauma (>12 weeks before inclusion) and ankle symptoms. The first question on history of ankle trauma was; ‘did you sustain an injury to the same ankle in the past?’ (yes or no); if yes, ‘how long ago?’ ((days) weeks, months or years) and ‘this was a: fracture, sprain, or otherwise, specify: … ‘. The second question regarding history of ankle trauma was whether the complaints arose directly after a trauma; if yes, ‘how long ago ?‘ ((days)weeks, months or years). Ankle symptoms included questions on duration (weeks, months or years). Participants were also asked to circle one or more of the following symptoms they experienced predominantly: pain, instability, functional loss and/or stiffness. Pain severity during rest and activity (0–10; no pain - worst pain imaginable) was measured using the numeric rating scale (NRS-11) [31]. The ankle osteoarthritis scale (AOS, 0–100; no pain/difficulty – worst pain imaginable/so difficult unable) was used to measure the severity of ankle pain and disability related to ankle OA [26]. The two subscales both contained nine questions about severity of pain and disability during various activities in the last two weeks.

### 2.4 Referral registry

The referring specialism (general practice, orthopaedic surgeon, surgeon, rheumatologist or internist) and reason for referral were extracted from the referral registry.

### 2.5 Radiography

Mortise and lateral views of the ankle(s) for which an X-ray was requested were taken from all participants. The X-rays were taken non-weight bearing unless weight bearing X-rays were requested. An experienced musculoskeletal radiologist (NK) systematically assessed the X-rays using a standardised scoring tool with good inter-observer reliability [3]. The examined features of radiographic OA were osteophytes, sclerosis, subchondral cysts and joint space narrowing. The degree of osteophytes and sclerosis were scored as absent, possibly, or evidently present (0–2) and subchondral cysts as absent or present (0 or 1). These abnormalities were scored for the medial and lateral malleolus; the tibiotalar surface of the tibia; the medial, lateral, subtalar and talonavicular surface of the talar bone; the subtalar surface of the calcaneus; and the talonavicular surface of the navicular bone. The scores were combined into the talocrural, subtalar and talonavicular joint. The degree of joint space narrowing was scored as absent, small, moderate or severe (0–3) for the talocrural, subtalar and talonavicular joint. An adapted version of the Kellgren and Lawrence (KL)-scale [3,29,32] combining the radiographic OA-features was scored as 0 ‘definite absence of radiographic changes of OA’, 1 ‘doubtful joint space narrowing and possible osteophytic lipping’, 2 ‘definite osteophytes and possible joint space narrowing’, 3 ‘moderate multiple osteophytes, definite narrowing of joint space, some sclerosis and possible deformity of bone ends’, or 4 ‘large osteophytes, marked narrowing of joint space, severe sclerosis and definite deformity of bone ends’ [32]. A KL-score 2 or higher was considered as radiographic OA [32]. For some analyses, features of radiographic OA and KL-scores were combined into a total KL-scale by taking the highest score in the talocrural, subtalar and/or talonavicular joint.

### 2.6 Data analysis

We assumed that the ankle (side) for which the radiograph was requested, was also the ankle with (most) complaints. The random number generator in Excel was used to include random sides in case of bilateral radiographs within one participant (n ​= ​68 (29.4%)).

Descriptive statistics were used to describe demographics, history and prevalence (number and percentage) of (features of) radiographic OA. Cases with more than 50% of the items of the AOS-pain or –disability subscale missing were not included in the analysis [33]. The mean and standard deviation (SD) were presented if variables were normally distributed, otherwise the median and inter-quartile range (IQR) was used.

Linear regression was used to assess the associations between degree of talocrural and talonavicular radiographic OA (KL-score 1 and ​≥ ​2 versus 0) and the primary outcomes severity of ankle symptoms (AOS-pain and AOS-disability). In addition, multiple linear regression was conducted with adjustment for age, sex, BMI and posttraumatic complaints. Measures of associations of each independent variable with the AOS-pain and AOS-disability were presented in unstandardized coefficients (B) with 95% confidence intervals (95% CI). To assess the associations between degree of talocrural and talonavicular radiographic OA and the secondary outcomes predominant symptoms (pain, functional loss, stiffness and instability), logistic regression was conducted with adjustment for age, sex, BMI and posttraumatic complaints. Measures of associations between the talocrural and talonavicular KL-score and the predominant symptoms after adjustment for age, sex, BMI and posttraumatic complaints were presented as Odds Ratios (OR) with corresponding 95% CIs. Data were analysed using IBM SPSS Statistics 25. *P*-values <0.05 were considered as statistically significant. An area-Proportional Venn diagram of three predominant symptoms (pain, functional loss, stiffness, or instability) with most frequent radiographic OA was drawn using the free software from eulerAPE [34].

## 3 Results

### 3.1 Participant characteristics

Of the total cohort population (N ​= ​893 participants), 231 (25.9%) participants had chronic ankle complaints and were included in the current study (Fig. 1). Demographics and details about symptoms for participants with different KL-scores are provided in Table 1.

**Table 1:** Participant characteristics.

	Total n ​= ​231	KL-score^b^ ​= ​0 n ​= ​104	KL-score^b^ ​= ​1 n ​= ​70	KL-score^b^ ≥2 n ​= ​57
Age (years) median (IQR)	52.53 (17.46)	48.07 (21.37)	54.55 (19.24)	55.87 (13.78)
Sex (female)^a^	125 (54.1%)	67 (65.7%)	31 (44.3%)	27 (48.2%)
BMI^b^ (kg/m^2^) median (IQR)^a^	27.74 (7.05)	27.58 (6.80)	27.38 (7.05)	28.46 (7.71)
Bilateral radiographs	68 (29.4%)	30 (28.8%)	26 (37.1%)	12 (21.1%)
Referrer^a^				
- General practitioner	133 (57.6%)	64 (61.5%)	36 (52.2%)	33 (57.8%)
- Orthopaedic surgeon	72 (31.2%)	32 (30.8%)	22 (31.9%)	18 (31.6%)
- Surgeon	6 (2.6%)	2 (1.9%)	2 (2.9%)	2 (3.5%)
- Rheumatologist/Internist	19 (8.2%)	6 (5.8%)	9 (13.0%)	4 (7.0%)
Post-traumatic complaints	148 (64.1%)	65 (62.5%)	42 (60.0%)	41 (71.9%)
Predominant symptoms^a^^,^^c^				
- Pain	189 (81.8%)	86 (92.5%)	60 (89.6%)	43 (84.3%)
- Feeling of instability	59 (25.5%)	27 (29.0%)	21 (31.3%)	11 (21.6%)
- Loss of function	42 (18.2%)	13 (14.0%)	14 (20.9%)	15 (29.4%)
- Stiffness	94 (40.7%)	38 (40.9%)	31 (46.3%)	25 (49.0%)
Pain (NRS^b^-11) median (IQR)				
- In rest^a^	3.00 (4.00)	4.00 (5.00)	3.00 (4.00)	3.00 (3.00)
- During activity^a^	7.00 (2.00)	7.00 (2.00)	7.00 (3.00)	7.00 (3.00)
Ankle Osteoarthritis Scale median (IQR)				
- pain (0–100)^a^	45.53 (26.04)	49.39 (24.10)	40.79 (22.91)	43.36 (21.95)
- disability (0–100)^a^	41.26 (37.53)	47.49 (42.53)	34.13 (28.59)	44.17 (43.50)

a Missing data: n = 3 (1.3%) for sex, n = 6 (2.6%) for BMI, n = 1 (0.4%) for referrer, n = 20 (8.7%) for predominant symptoms, n = 9 (3.9%) for pain in rest, n = 11 (4.8%) for pain during activity, n = 42 (18.2%) for ankle osteoarthritis scale – pain (n = 18, n = 10, n = 14 for KL-score 0, 1 and ≥ 2, respectively), n = 53 (22.9%) for ankle osteoarthritis scale – disability (n = 24, n = 15, n = 14 for KL-score 0, 1 and ≥ 2, respectively).

b KL, Kellgren & Lawrence; BMI, body mass index; NRS, numeric rating scale.

c Symptoms add up over 100%.

The median (±IQR) age of the participants was 52.53 (±17.46), 54.1% were women and the median BMI (±IQR) was 27.7 (7.1) kg/m^2^. General practitioners referred most participants (57.6%). The majority of participants (64.1%) had posttraumatic complaints. The median (±IQR) AOS-score was 45.53 (±26.04) for pain, and 41.26 (±37.53) for disability. The most prevailing predominant symptom was pain (81.8%), followed by stiffness (40.7%), feeling of instability (25.5%) and functional loss (18.2%).

Prevalence of radiographic OA features are presented in Table 2. In total (talocrural, subtalar and talonavicular joint combined) 24.7% had radiographic ankle OA. None of the radiographic OA occurred in the subtalar joint.

**Table 2:** Prevalence of features of radiographic ankle osteoarthritis.

Abnormality n (%)	Talocrural joint	Subtalar joint	Talonavicular joint^a^	Total N ​= ​231
Osteophyte				
None	120 (51.9%)	227 (98.3%)	151 (65.4%)	96 (41.6%)
Possibly	68 (29.4%)	3 (1.3%)	43 (18.6%)	69 (29.9%)
Evident	43 (18.6%)	1 (0.4%)	36 (15.6%)	66 (28.6%)
Subchondral cyst				
Absent	224 (97.0%)	231 (100.0%)	224 (97.0%)	219 (94.8%)
Present	7 (3.0%)	0 (0%)	6 (2.6%)	12 (5.2%)
Sclerosis				
None	213 (92.2%)	223 (96.5%)	120 (51.9%)	113 (48.9%)
Possibly	14 (6.1%)	8 (3.5%)	106 (45.9%)	110 (47.6%)
Evident	4 (1.7%)	0 (0.0%)	4 (1.7%)	8 (3.5%)
Joint space narrowing				
None	178 (77.1%)	228 (98.7%)	181 (78.4%)	152 (65.8%)
Possibly	37 (16.0%)	3 (1.3%)	37 (16.0%)	54 (23.4%)
Moderate	10 (4.3%)	0 (0.0%)	12 (5.2%)	19 (8.2%)
Severe	6 (2.6%)	0 (0.0%)	0 (0.0%)	6 (2.6%)
KL-score^b^				
Normal	127 (55.0%)	227 (98.3%)	154 (66.7%)	104 (45.0%)
Grade 1	67 (29.0%)	4 (1.7%)	48 (20.8%)	70 (30.3%)
Grade 2	30 (13.0%)	0 (0.0%)	26 (11.3%)	48 (20.8%)
Grade 3	4 (1.7%)	0 (0.0%)	2 (0.9%)	6 (2.6%)
Grade 4	3 (1.3%)	0 (0.0%)	0 (0.0%)	3 (1.3%)

a Missing data: n = 1 (0.4%) for talonavicular joint.

b KL, Kellgren & Lawrence.

### 3.2 Association between the degree of radiographic ankle OA and severity of ankle pain and disability

Talocrural KL grade 1 vs. 0, but not ≥2 vs. 0, was associated with lower AOS disability and pain scores (adjusted B ​= ​−10.08 (95%CI -18.18;-1.97) and −6.30 (95% CI -12.23; −0.36), respectively). Radiographic OA was not associated with the AOS disability and pain score in the talocrural (adjusted B −2.05 (95% CI -12.12; 8.01) and −1.89 (95% CI -9.54; 5.77), respectively) and talonavicular joint (adjusted B ​= ​−10.23 (95%CI -22.35; 1.88) and −5.93 (95% CI -14.84; 2.98), respectively) (Table 3).

**Table 3:** Association between radiographic ankle osteoarthritis and the Ankle Osteoarthritis Score.

Parameter	Adjusted B (95% CI)^a^	p-value	Adjusted B (95% CI)^a^	p-value
	Talocrural		Talonavicular	
**Ankle Osteoarthritis Score – Disabilityn ​= ​175**				
Age (years)	0.28 (0.01; 0.55)	0.042∗	0.32 (0.05; 0.59)	0.020∗
Sex (female)	8.86 (1.77; 15.95)	0.015∗	7.96 (0.65; 15.28)	0.033∗
Body Mass Index (kg/m^2^)	0.19 (−0.50; 0.88)	0.583	0.20 (−0.49; 0.89)	0.568
Posttraumatic	−4.82 (−12.42; 2.78)	0.212	−5.12 (−12.71; 2.48)	0.185
KL^a^-score (0 ​= ​reference)				
- Grade 1	−10.08 (−18.18; −1.97)	0.015∗	−7.44 (−16.54; 1.66)	0.108
- Grade ≥2	−2.05 (−12.12; 8.01)	0.688	−10.23 (−22.35; 1.88)	0.097
**Ankle Osteoarthritis Score - Pain n ​= ​185**				
Age (years)	−0.06 (−0.26; 0.15)	0.577	−0.06 (−0.26; 0.15)	0.589
Sex (female)	8.56 (3.30; 13.82)	0.002∗	8.40 (3.01; 13.79)	0.002∗
Body Mass Index (kg/m^2^)	0.65 (0.13; 1.16)	0.015∗	0.69 (0.16; 1.21)	0.011∗
Posttraumatic	−6.13 (−11.69; −0.57)	0.031∗	−6.23 (−11.79; −0.67)	0.028∗
KL^a^-score (0 ​= ​ref)				
- Grade 1	−6.30 (−12.23; −0.36)	0.038∗	−2.68 (−9.27; 3.91)	0.423
- Grade ≥2	−1.89 (−9.54; 5.77)	0.627	−5.93 (−14.84; 2.98)	0.191

a KL, Kellgren & Lawrence; B, unstandardized regression coefficients; CI, Confidence Interval.

### 3.3 Association between degree of radiographic ankle OA and predominant symptoms

**Fig. 2:**
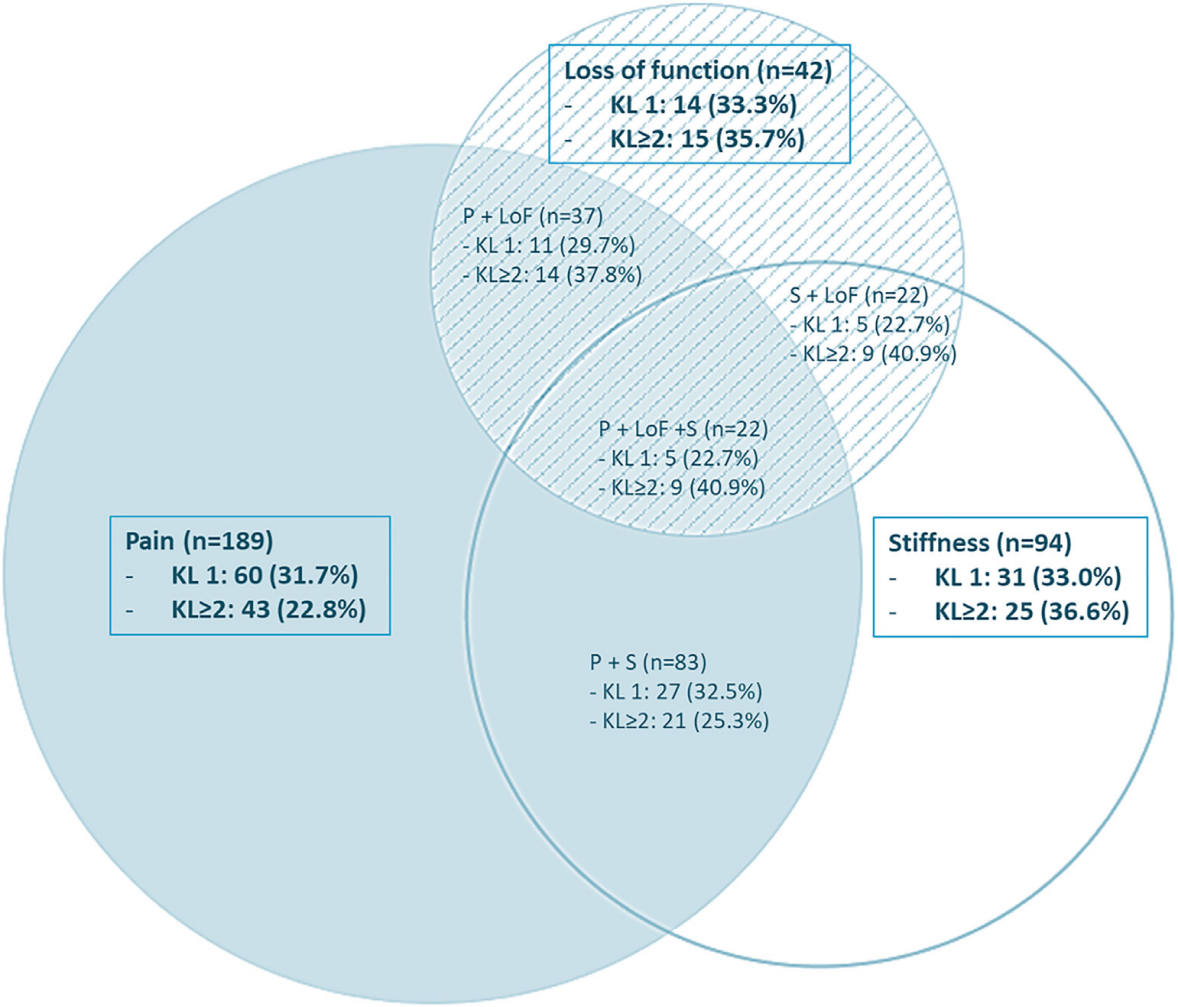
VENN-diagram of predominant symptoms. (a). VENN-diagram showing the number of participants with pain, loss of function and/or stiffness as predominant symptom. (b). Blue = pain (P), striped = loss of function (LoF), white = stiffness (S). (c). In each (overlapping) symptom, the percentage of participants with an abnormal Kellgren and Lawrence (KL) score is presented. (For interpretation of the references to color in this figure legend, the reader is referred to the Web version of this article.)

Radiographic OA occurred in 11 out of 59 (18.6%) participants with instability as one of the predominant symptoms. The percentage of radiographic OA was 22.8% in participants with pain, 36.6% in participants with stiffness and 35.7% in participants with functional loss as predominant symptom (Fig. 2). The percentage of radiographic OA was highest in participants with both functional loss and stiffness (40.9%) as predominant symptoms.

Radiographic talocrural OA was associated with functional loss as predominant symptom (adjusted OR 3.26 (95%CI 1.31; 8.11)). A similar direction, but no statistically significant association was seen for radiographic talonavicular OA and stiffness as predominant symptom (adjusted OR 2.63 (95%CI 0.97; 7.15)). Degree of radiographic ankle OA was not associated with instability and pain as predominant symptoms (Table 4).

**Table 4:** Association between predominant symptoms and radiographic ankle osteoarthritis.

KL^b^-score	Predominant symptom	Absent n (%)	Present n (%)	Adjusted OR^b^ (95% CI^b^)^a^
Talocrural n ​= ​204				

Normal	Pain	10 (45.5%)	103 (54.5%)	Reference
Grade 1	(n ​= ​189, 81.8%)	6 (27.3%)	57 (30.2%)	1.12 (0.37; 3.38)
≥ Grade 2		6 (27.3%)	29 (15.3%)	0.62 (0.18; 2.15)
Normal	Functional loss	94 (55.6%)	19 (45.2%)	Reference
Grade 1	(n ​= ​42, 18.2%)	53 (31.4%)	10 (23.8%)	1.00 (0.42; 2.37)
≥ Grade 2		22 (13.0%)	13 (31.0%)	**3.26 (1.31; 8.11)**^c^
Normal	Stiffness	65 (55.6%)	48 (51.1%)	Reference
Grade 1	(n ​= ​94, 40.7%)	33 (28.2%)	30 (31.9%)	1.19 (0.62; 2.26)
≥ Grade 2		19 (16.2%)	16 (17.0%)	0.92 (0.40; 2.09)
Normal	Instability	80 (52.6%)	33 (55.9%)	Reference
Grade 1	(n ​= ​59, 25.5%)	47 (30.9%)	16 (27.1%)	0.98 (0.47; 2.03)
≥ Grade 2		25 (16.4%)	10 (16.9%)	1.25 (0.51; 3.09)
Talonavicular n ​= ​203				
Normal	Pain	13 (59.1%)	125 (66.5%)	Reference
Grade 1	(n ​= ​188, 81.4%)	6 (27.3%)	42 (22.3%)	0.92 (0.31; 2.73)
≥ Grade 2		3 (13.6%)	21 (11.2%)	1.13 (0.22; 5.85)
Normal	Functional loss	113 (66.9%)	25 (61.0%)	Reference
Grade 1	(n ​= ​41, 17.7%)	36 (21.3%)	12 (29.3%)	1.33 (0.57; 3.10)
≥ Grade 2		20 (11.8%)	4 (9.8%)	0.88 (0.26; 2.93)
Normal	Stiffness	78 (67.2%)	60 (63.8%)	Reference
Grade 1	(n ​= ​94, 40.7%)	30 (25.9%)	18 (19.1%)	0.82 (0.40; 1.67)
≥ Grade 2		8 (6.9%)	16 (17.0%)	2.63 (0.97; 7.15)
Normal	Instability	95 (62.9%)	43 (72.9%)	Reference
Grade 1	(n ​= ​59, 25.5%)	34 (22.5%)	14 (23.7%)	1.02 (0.47; 2.22)
≥ Grade 2		22 (14.6%)	2 (3.4%)	0.28 (0.06; 1.31)

a adjusted for age, sex, bmi and trauma.

b KL, Kellgren & Lawrence; OR, Odds ratio; CI, confidence interval.

c Odds ratios and confidence intervals in bold are statistically significant.

## 4 Discussion

Radiographic ankle OA was not associated with severity of ankle pain and disability measured with the AOS. Though, radiographic talocrural OA was associated with functional loss as predominant symptom and, though not statistically significant, a similar direction was seen for radiographic talonavicular OA and stiffness as predominant symptom. Degree of radiographic ankle OA was not associated with both pain and instability as predominant symptom.

The absence of an association between radiographic ankle OA and severity of complaints, and the negative association between doubtful radiographic ankle OA (KL grade 1 vs 0) and severity of disability and pain measured with the AOS were surprising. However, the magnitude of the associations between KL grade 1 vs 0 and AOS pain and disability scores is small. Moreover, other radiographic abnormalities, i.e. fractures, calcifications, osteochondral defects, signs of anterior impingement, intraarticular hydrops, corpus liberum or soft tissue swelling might also have influenced the AOS-score. Consequently, we computed the multiple linear regression model as an additional sensitivity analysis in a population without any of the other radiographic abnormalities (Supplementary material; table A), but the direction of the association between doubtful radiographic ankle OA and the AOS remained unchanged. Therefore, we are convinced that the other radiographic abnormalities did not cause this seemingly contradictory finding. In line with other studies using the AOS-score, items on pain during the use of braces/insoles were frequently missing [33,35]. To make sure that including the two items on braces/insoles did not create any bias, we repeated the analyses on the association between radiographic ankle OA and AOS-pain subscale excluding those items. However, this did not change our results (Supplementary material; table B). We did not find a positive direction of associations either between the separate AOS items and separate features of radiographic OA (Supplementary material; table C and D). This finding is contrary to previous studies, that found radiographic ankle OA to be associated with symptoms, measured by the AOFAS ankle-hindfoot and VAS pain score in a population with posttraumatic ankle OA [14], but also by the AOS in populations with end-stage ankle OA [13] and isolated unilateral ankle OA [26]. Our study included only few participants (3.9%) with a KL-score >2, compared to 26.7% in the study population with posttraumatic ankle OA [14]. Therefore, the AOS might be unsuitable as a tool to determine the presence of radiographic ankle OA in a population with relatively low degrees of radiographic ankle OA.

To our knowledge, this is the first study to assess the association between radiographic ankle OA and different types of symptoms. Radiographic talocrural OA was associated with the presence of functional loss as predominant symptom. No association was found between radiographic OA and pain as predominant symptom, but this is likely due to the low number of participants in our population reporting ‘no pain’. Moreover, a positive trend was seen between radiographic talonavicular OA and stiffness as predominant symptom, although this was not statistically significant. Associations between radiographic OA and stiffness were found in the knee [17,18]. Moreover, stiffness is known to be associated with osteophytes and disk space narrowing in people with low back pain and a hip and/or knee pain history [36]. In additional analysis we did not find stiffness as predominant symptom to be associated with the AOS, while functional loss as predominant symptom was associated with both the AOS pain and disability sub scores (Supplementary material table E). However, the AOS does not include the assessment of stiffness [13,26]. This is in contrast with for example the Knee injury and OA Outcome Score (KOOS) and Western Ontario [37] and McMaster Universities Arthritis Index (WOMAC) [38] for knee and/or hip OA. Given the results of the present study and as stiffness is included in evidence-based diagnostic criteria for clinical knee OA, i.e. the NICE, EULAR and ACR [[21], [22], [23],^39^], we think that stiffness also could be considered as an important item for the definition of clinical ankle OA.

Our results show that radiographic OA in the talonavicular joint and the talocrural joint were associated with different predominant ankle symptoms. So far, most studies that assessed the association between radiographic OA and ankle symptoms only examined the talocrural joint [10,14], or examined the talonavicular joint as a component of ‘medial midfoot’ OA [[40], [41], [42]] with some finding an association with foot pain [41], but others not [40]. As the subtalar and talonavicular joint are usually visible on lateral ankle radiographs, and radiologists will report on existing radiographic OA in these joints in clinical practice, it is important to understand their relationship with ankle symptoms. However, in our study population, no radiographic OA was found in the subtalar joint, which might be due to a lack of use of the specific Broden's view [3,4] in clinical practice. As radiographic talocrural and talonavicular OA are related to different symptoms, future research is warranted to examine clinical and demographic differences between talocrural and talonavicular OA and OA in other foot joints.

### 4.1 Strengths and limitations

This is the first study to assess the association between radiographic ankle OA and ankle symptoms in a population referred due to chronic ankle complaints. This study population reflects a patient population in which it is important to know which symptoms are associated with radiographic ankle OA.

Although our sample was sufficiently large, the number of participants in our population with radiographic talonavicular and talocrural OA was relatively small, increasing the risk of a type II Error. We did not choose to collapse KL-scores, or to combine the talocrural and talonavicular joint to increase power, because that would cause an undesirable loss of information. However, analysing the association between different symptoms and radiographic talocrural and talonavicular OA apart, might have led to a type I error by using multiple repeated tests. We did not use a Bonferroni-correction to adjust for this. Due to the cross-sectional study design, fluctuations in symptoms, and therefore a possible association between severity of symptoms and degree of radiographic OA might have been missed.

Participants with missing values for the AOS-disability had a significantly higher pain-score (NRS-11) in rest compared to the other participants, though no differences were seen in KL-scores. Therefore, we think that the association between AOS and degree of radiographic ankle OA might be slightly biased.

The questionnaires did not include any questions about the side of (most) complaints, hence we choose to randomly select a side in case of bilateral radiographs within one participant. We only collected information about presence of stiffness. Details about duration and morning stiffness may be included in future studies on symptoms associated with ankle OA. As earlier research showed that higher AOS-scores were associated with arthritis in general [26] and higher AOS-pain scores with musculoskeletal problems of other joints [5], it would have been informative to include questions on musculoskeletal comorbidities in the questionnaire.

As the standard hospital policy is to take the ankle radiographs in non-weight bearing unless specifically requested otherwise, the degree of joint space narrowing might have been underestimated. Unfortunately, we could not retrieve which radiographs were taken weight bearing.

## 5 Conclusion

This study examined the relationship between degree of radiographic ankle OA and severity and type of ankle symptoms. The degree of radiographic ankle OA is not associated with severity of pain and disability measured by the AOS in a population with chronic ankle complaints referred for an ankle X-ray. Moreover, the results show that radiographic talocrural OA is associated with functional loss as predominant symptom and, though not statistically significant, a similar direction was seen for radiographic talonavicular OA and stiffness as predominant symptom. Given our results, we suggest not to use the AOS as a diagnostic tool for ankle OA in a population with chronic ankle complaints, but recommend functional loss and stiffness as candidates for future diagnostic criteria for ankle OA.

## Author contributions

Marienke van Middelkoop and Nienke Katier were responsible for the conception and design of the study. Nienke Katier and Jeanette van Vooren performed and supervised the data acquisition. Sabine Kloprogge, Marienke van Middelkoop, Nienke Katier and Sita Bierma-Zeinstra performed analysis and interpretation of the data. Sabine Kloprogge drafted the manuscript, which was critically revised by all authors. All authors approved the final version of the manuscript.

## Role of the funding source

This work was supported by the General Practice department of the Erasmus MC Medical University Centre and the radiology department of the Albert Schweitzer Hospital.

## Declaration of competing interest

None declared.
