# Supplementary material for: Is radiographic osteoarthritis associated with pain and disability of the ankle?
Source: Osteoarthr Cartil Open. 2023 Jul 7;5(3):100383. doi: 10.1016/j.ocarto.2023.100383 (PMC10371817; doi:10.1016/j.ocarto.2023.100383)
Supplement: Multimedia component 1 [file mmc1.docx]

Supplementary material

# Table A: Association between radiographic ankle osteoarthritis and the Ankle Osteoarthritis Score; without other radiographic abnormalities*

| **Parameter** | **Adjusted B (95% CI)** | **p-value** | **Adjusted B (95% CI)** | **p-value** |
| --- | --- | --- | --- | --- |
|  | **Talocrural** | | **Talonavicular** | |
| **Ankle Osteoarthritis Score – Disability**  n=97 | | | | |
| **KL-score** (0=reference)  - Grade 1  - Grade ≥2 | -13.90 (-24.81; -2.99)  1.39 (-16.27; 19.04) | 0.013*  0.876 | -13.88 (-28.13; 0.37)  -16.78 (-36.91; 3.34) | 0.056  0.101 |
| **Ankle Osteoarthritis Score - Pain**  n=101 | | | | |
| **KL-score** (0=ref)  - Grade 1  - Grade ≥2 | -8.07 (-16.22; 0.09)  -2.02 (-16.43; 12.39) | 0.052  0.781 | -6.83 (-16.68; 3.01)  -10.70 (-26.10; 4.69) | 0.172  0.171 |

KL, Kellgren & Lawrence; B, unstandardized regression coefficients; CI, Confidence Interval

Analyses were adjusted for age, sex, body mass index and posttraumatic complaints.

* Other radiographic abnormalities excluded from analyses: Fractures, calcifications, osteochondral defects, anterior impingement, intra-articular hydrops, intra-articular corpus liberum, soft tissue swelling.

# Table B: Association between radiographic ankle osteoarthritis and the Ankle Osteoarthritis Score - Pain; without brace/insole items

| **Parameter** | **Adjusted B (95% CI)** | **p-value** | **Adjusted B (95% CI)** | **p-value** |
| --- | --- | --- | --- | --- |
|  | **Talocrural** | | **Talonavicular** | |
| **Ankle Osteoarthritis Score - Pain**  n=188 | | | | |
| **KL-score** (0=ref)  - Grade 1  - Grade ≥2 | -6.38 (-12.21; -0.54)  -3.19 (-10.75; 4.36) | 0.032*  0.405 | -2.35 (-8.85; 4.15)  -5.66 (-14.45; 3.14) | 0.476  0.206 |

KL, Kellgren & Lawrence; B, unstandardized regression coefficients; CI, Confidence Interval

Analyses were adjusted for age, sex, body mass index and posttraumatic complaints.

# Table C: Association between radiographic ankle osteoarthritis and the Ankle Osteoarthritis Score; separate items

| **Parameter** | **Adjusted B (95% CI)** | **P-value** | **Adjusted B (95% CI)** | | **P-value** |
| --- | --- | --- | --- | --- | --- |
|  | **Talocrural** | | | **Talonavicular** | |
| **AOS – pain; as its worst** (n=167) | | | | | |
| - KL Grade 1  - KL Grade ≥2 | -1.70 (-9.53; 6.12)  -1.90 (-11.43; 7.63) | .668  .694 | 1.80 (-6.74; 10.33)  1.28 (-10.14; 12.71) | | .678  .825 |
| **AOS – pain; before you get up in the morning** (n=185) | | | | | |
| - KL Grade 1  - KL Grade ≥2 | -2.70 (-11.39; 6.00)  -9.57 (-20.43; 1.30) | .541  .084 | -2.81 (-12.39; 6.78)  -6.58 (-19.36; 6.20) | | .564  .311 |
| **AOS – pain; when you walked barefoot** (n=186) | | | | | |
| - KL Grade 1  - KL Grade ≥2 | -6.48 (-14.62; 1.67)  1.65 (-8.81; 12.11) | .118  .756 | -3.62 (-12.58; 5.35)  -6.12 (-18.32; 6.07) | | .427  .323 |
| **AOS – pain; when you stood barefoot** (n=184) | | | | | |
| - KL Grade 1  - KL Grade ≥2 | -4.81 (-13.09; 3.47)  -6.08 (-16.80; 4.63) | .253  .264 | 2.63 (-6.44; 11.70)  -9.19 (-21.73; 3.36) | | .568  .150 |
| **AOS – pain; when you walked wearing shoes** (n=189) | | | | | |
| - KL Grade 1  - KL Grade ≥2 | -10.80 (-18.30; -3.29)  -2.73 (-12.41; 6.94) | .005*  .578 | -8.25 (-16.62; 0.12)  -9.34 (-20.86; 2.19) | | .053  .112 |
| **AOS – pain; when you stood wearing shoes** (n=182) | | | | | |
| - KL Grade 1  - KL Grade ≥2 | -8.26 (-16.32; -0.21)  -3.45 (-14.12; 7.22) | .044*  .524 | -2.04 (-11.03; 6.95)  -4.41 (-16.76; 7.94) | | .655  .482 |
| **AOS – pain; when you walked wearing shoe inserts or braces** (n=91) | | | | | |
| - KL Grade 1  - KL Grade ≥2 | -6.51 (-19.96; 6.95)  10.80 (-7.57; 29.18) | .339  .246 | -7.61 (-22.11; 6.90)  -9.21 (-27.55; 9.13) | | .300  .321 |
| **AOS – pain; when you stood wearing shoe inserts or braces** (n=88) | | | | | |
| - KL Grade 1  - KL Grade ≥2 | -6.44 (-20.05; 7.18)  7.51 (-11.69; 26.71) | .350  .439 | -0.40 (-15.18; 14.37)  -9.23 (-27.70; 9.24) | | .975  .323 |
| **AOS – pain; at the end of the day** (n=192) | | | | | |
| - KL Grade 1  - KL Grade ≥2 | -9.11 (-17.34; -0.88)  -6.24 (-16.57; 4.08) | .030*  .234 | -3.41 (-12.61; 5.79)  -2.63 (-14.86; 9.59) | | .466  .671 |
| **AOS – disability; walking around the house** (n=175) | | | | | |
| - KL Grade 1  - KL Grade ≥2 | -8.63 (-17.63; 0.36)  2.15 (-8.50; 12.80) | .060  .691 | -7.49 (-17.44; 2.46)  -9.16 (-22.11; 3.79) | | .139  .164 |
| **AOS – disability; walking outside on uneven ground** (n=170) | | | | | |
| - KL Grade 1  - KL Grade ≥2 | -10.97 (-21.76; -0.17)  3.44 (-9.95; 16.84) | .046*  .613 | -2.82 (-14.95; 9.31)  -7.88 (-24.20; 8.43) | | .646  .341 |
| **AOS – disability; walking four blocks or more** (n=172) | | | | | |
| - KL Grade 1  - KL Grade ≥2 | -12.85 (-23.37; -2.33)  3.03 (-9.69; 15.75) | .017*  .639 | -6.41 (-18.19; 5.37)  -12.20 (-28.15; 3.75) | | .284  .133 |
| **AOS – disability; climbing stairs** (n=173) | | | | | |
| - KL Grade 1  - KL Grade ≥2 | -12.93 (-22.98; -2.87)  -7.50 (-19.91; 4.90) | .012*  .234 | -7.01 (-18.12; 4.11)  -13.46 (-28.32; 1.40) | | .215  .075 |
| **AOS – disability; descending stairs** (n=176) | | | | | |
| - KL Grade 1  - KL Grade ≥2 | -10.89 (-21.07; -0.72)  -5.20 (-17.88; 7.47) | .036*  .419 | -8.73 (-20.16; 2.70)  -11.76 (-27.34; 3.83) | | .133  .138 |
| **AOS – disability; standing on tip toes** (n=159) | | | | | |
| - KL Grade 1  - KL Grade ≥2 | -11.56 (-22.76; -0.37)  -0.43 (-13.98; 13.12) | .043*  .950 | 1.79 (-10.57; 14.16)  -2.07 (-19.07; 14.93) | | .775  .810 |
| **AOS – disability; getting out of a chair** (n=178) | | | | | |
| - KL Grade 1  - KL Grade ≥2 | -8.42 (-17.83; 1.00)  -8.88 (-20.79; 3.04) | .079  .143 | -6.71 (-17.14; 3.71)  -5.21 (-19.70; 9.29) | | .206  .479 |
| **AOS – disability; climbing up or down curbs** (n=171) | | | | | |
| - KL Grade 1  - KL Grade ≥2 | -9.11 (-18.95; 0.74)  -7.14 (-19.48; 5.20) | .070  .255 | -9.65 (-20.43; 1.13)  -15.67 (-30.15; -1.20) | | .079  .034* |
| **AOS – disability; walking fact or running** (n=164) | | | | | |
| - KL Grade 1  - KL Grade ≥2 | -5.47 (-16.29; 5.34)  8.82 (-4.76; 22.40) | .319  .201 | -8.34 (-19.91; 3.23)  -18.58 (-35.06; -2.10) | | .157  .027* |

KL, Kellgren & Lawrence; B, unstandardized regression coefficients; CI, Confidence Interval

KL-score 0 = reference

Analyses were adjusted for age, sex, body mass index and posttraumatic complaints.

# Table D: Association between radiographic ankle osteoarthritis and the Ankle Osteoarthritis Score; separate features of radiographic OA

| **Parameter** | **Adjusted B (95% CI)** | **P-value** | **Adjusted B (95% CI)** | | **P-value** |  |
| --- | --- | --- | --- | --- | --- | --- |
|  | **Talocrural** | | | **Talonavicular** | | |
| **AOS – pain** | | | | | | |
| **Osteophytes** n=185 | -1.14 (-4.69; 2.41) | .528 | -2.92 (-6.61; 0.77) | | .120 |  |
| **Sclerosis** n=185 | 3.90 (-3.21; 11.01) | .280 | -1.51 (-6.56; 3.54) | | .556 |  |
| **Joint space narrowing** n=185 | -5.54 (-11.95; 0.86) | .089 | -4.65 (-9.65; 0.36) | | .068 |  |
| **AOS – disability** | | | | | | |
| **Osteophytes** n=175 | -2.32 (-7.05; 2.41) | .334 | -6.07 (-11.13; -1.02) | | .019* |  |
| **Sclerosis** n=175 | 9.36 (-2.99; 21.70) | .136 | -0.20 (-7.52; 7.12) | | .956 |  |
| **Joint space narrowing** n=175 | -3.60 (-12.19; 4.99) | .409 | -4.49 (-11.47; 2.49) | | .206 |  |

1. Analyses with subchondral cysts were not performed, as the presence was below 10.
2. Analyses were adjusted for age, sex, body mass index and posttraumatic complaints.

# Table E: Association between predominant symptoms and the Ankle Osteoarthritis Score

| **Pearson Correlation** | **AOS-pain** | **AOS- disability** | **Predominant symptom- stiffness** | **Predominant symptom- functional loss** |
| --- | --- | --- | --- | --- |
| **AOS-pain** | 1 | .537^**^ | .061 | .125 |
| **AOS-disability** | .537^**^ | 1 | .130 | .352^**^ |
| **Predominant symptom-stiffness** | .061 | .130 | 1 | .079 |
| **Predominant symptom- functional loss** | .125 | .352^**^ | .079 | 1 |

** Correlation is significant at the 0.01 level (2-tailed)
